# Role of CGRP pathway polymorphisms in migraine: a systematic review and impact on CGRP mAbs migraine therapy

**DOI:** 10.1186/s10194-021-01295-7

**Published:** 2021-07-30

**Authors:** Damiana Scuteri, Maria Tiziana Corasaniti, Paolo Tonin, Pierluigi Nicotera, Giacinto Bagetta

**Affiliations:** 1 Pharmacotechnology Documentation and Transfer Unit, Preclinical and Translational Pharmacology, Department of Pharmacy, Health and Nutritional Sciences, University of Calabria, 87036 Rende, Italy; 2 Regional Center for Serious Brain Injuries, S. Anna Institute, Crotone, Italy; 3 Department of Health Sciences, University “Magna Graecia” of Catanzaro, 88100 Catanzaro, Italy; 4 German Center for Neurodegenerative Diseases (DZNE), Bonn, Germany

**Keywords:** polymorphisms, SNPs, methylation, epigenetic, migraine, CGRP, CALC A, RAMP 1, CLR, RCP, CALCRL, AMYLIN-1, systematic review

## Abstract

**Background:**

the interest of clinical reaseach in polymorphisms and epigenetics in migraine has been growing over the years. Due to the new era of preventative migraine treatment opened by monoclonal antibodies (mAbs) targeting the signaling of the calcitonin-gene related peptide (CGRP), the present systematic review aims at identifying genetic variants occurring along the CGRP pathway and at verifying whether these can affect the clinical features and the course of disease and the responsiveness of patients to therapy.

**Methods:**

the literature search has been conducted consulting the most relevant scientific databases, i.e. PubMed/MEDLINE, Scopus, Web of Science, the Human Genome Epidemiology (HuGE) Published Literature database (Public Health Genomics Knowledge Base) and Clinicaltrials.gov from database inception until April 1, 2021. The process of identification and selection of the studies included in the analysis has followed the PRISMA (Preferred Reporting Items for Systematic reviews and Meta-Analyses) criteria for systematic reviews and meta-analyses and the guidance from the Human Genome Epidemiology Network for reporting gene-disease associations.

**Results:**

the search has retrieved 800 results, among which only 7 studies have met the eligibility criteria for inclusion in the analysis. The latter are case-control studies of genetic association and an exploratory analysis and two polymorphisms have been detected as the most recurring: the rs3781719 (T > C) of the CALC A gene encoding CGRP and the rs7590387 of the gene encoding the receptor activity-modifying protein (RAMP) 1 (C > G). Only one study assessing the methylation pattern with regard to CGRP pathway has been found from the search. No genetic association studies investigating the possible effect of genetic variants affecting CGRP signaling on the responsiveness to the most recent pharmacological approaches, i.e. anti-CGRP(R) mAbs, gepants and ditans, have been published. According to the Human Genome Epidemiology (HuGE) systematic reviews and meta-analyses risk-of-bias score for genetic association studies, the heterogeneity between and across studies and the small sample size do not allow to draw conclusions and prompt future studies.

**Conclusions:**

adequately powered, good quality genetic association studies are needed to understand the impact of genetic variants affecting the pathway of CGRP on migraine susceptibility and clinical manifestation and to predict the response to therapy in terms of efficacy and safety.

## Background

### Rationale and objective

**Fig. 1:**
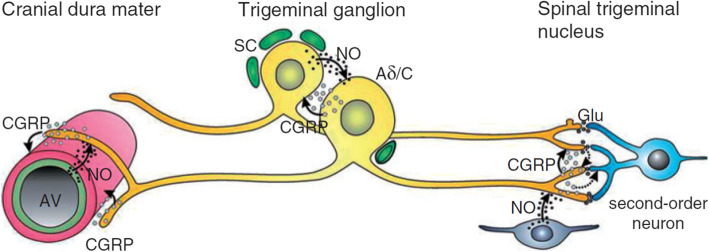
The Calcitonin-gene related peptide (CGRP)-signaling in the trigeminovascular system. The CGRP released from perivascular afferents in the dura, causes dilation of arterial vessels (AV). Nitric oxide (NO) from the vascular endothelium facilitates CGRP release. The CGRP signals to trigeminal ganglion neurons (Aδ/C) CGRP-ergic receptors inducing facilitation of nociceptive transmission to second-order neurons possibly by increasing the release of the excitatory neurotransmitter glutamate (Glu) from neighboring primary afferent terminals. CGRP may also signal directly to second-order neurons (dotted arrows). Adapted with permission from [11].

Migraine is a primary headache disorder defined as a prevalent neurologic disease characterized by headaches that can occur with or without aura, consisting of transient focal neurological symptoms (visual, sensory, speech and/or language, motor, brainstem and retinal) that usually precede by hours or days, or sometimes accompany, the headache [1]. The social worldwide burden of migraine is noteworthy since it ranks the sixth most prevalent disease and the second cause of disability worldwide [2], accounting for around 7 % of all-cause Years Lived with Disability (YLD) and for 72 % of all YLDs associated to neurological disorders [3]. In fact, according to report from the Global Burden of Disease Headache in 2018, 14.4 % of the global population suffers from migraine making of it the global second leading cause of disability [4]. Migraine belongs to the category of chronic diseases since it is characterized by episodic manifestations (CDEM) [5] that can undergo chronification in the process of *clinical transformation and progression* [6]. The prevention of the episodic attacks is fundamental to avoid chronification. The nociceptors from the *dura mater* and periorbital skin project [7] to second-order neurons in the trigeminal *nucleus caudalis* [8, 9], that can be subjected to sensitization as third-order neurons mainly in the *pulvinar* of the thalamus. The sensitization of the latter induces cutaneous allodynia, cephalic at the beginning and generalized or extracephalic at later stage [8, 9] and it is involved in chronification. The vasodilatory neuropeptides are remarkably implicated in the latter dural neurogenic inflammation. Among these, calcitonin-gene related peptide (CGRP) is the the most important player responsible for clinically relevant vasodilation acting on its receptor in the trigeminal ganglion [10] (Fig. 1).

In particular, the α-CGRP encoded by the CALC A (or CALC I) gene is involved in the pathogenesis of migraine [12]. The effects of CGRP are mediated by its interaction with the CGRP receptor, a Gα_s_ protein-coupled receptor formed by the calcitonin receptor-like receptor (CLR), the receptor activity-modifying protein (RAMP) 1 and the receptor component protein (RCP) [13].CGRP requires also the fusion protein of the extracellular domains of human G protein-coupled receptor calcitonin receptor-like receptor CALCRL to activate the downstream signaling that ends with vasodilation [14]. Apart from this canonical receptor, CGRP signal transduction is mediated by the second receptor that is the human amylin subtype 1 receptor (AMY1). The genes encoding the latter molecules responsible for CGRP-induced signaling are subjected to genetic variants influencing their activities. Specific anti-migraine drugs for acute treatment of attacks, e.g. triptans, are agonists of 5-HT1B, 5-HT1D and 5-HT1F that finally inhibit CGRP release during migraine attacks [12]. Furthermore, novel therapeutic and preventative approaches target the CGRP signaling: these are the gepants, antagonists of CGRP receptor, and the anti-CGRP(R) monoclonal antibodies (mAbs) [15]. Although the anti-CGRP(R) mAbs are the first specific preventive therapy which can provide pain relief to difficult-to-treat patients [12], some 40 % of the latter are non responders [16]. Apart from the monogenic forms of migraine and the evidence of rare pathologic genetic variants, several single-nucleotide polymorphisms (SNPs) have been associated with differences in migraine susceptibility, clinical features and response to treatment. For instance, genes related to vascular modifications and cardiovascular diseases, e.g. SNPs of the gene OMIM encoding the angiotensin converting enzyme (ACE) or SNPs of the methylenetetrahydrofolate reductase (MTHFR) genetic variants have been implicated in the susceptibility to migraine and aura and in the frequency of migraine attacks. The human leukocyte antigens (HLA) have been associated in heredity for migraine [17] and the HLA Class II DR2 antigen has been found to have a protective role toward migraine with aura [18, 19]. Interestingly, some SNPs have been associated with responsiveness to drugs and, thus, to tendency to chronification with overuse and medications overuse headache (MOH) [20]. Thus, in the future genetic profiling will be fundamental to foresee the efficacy and safety of therapy, depending on individual genetic variability [21, 22] and to design new drugs tailored on each patient’s genetics [23]. For example, the SNP C825TC of rs5443 in the gene GNB3 coding the G protein β3 subunit in the signaling of 5HT_1B/1D_ is a common genetic variant implicated in the rate of good responsiveness to triptans [24, 25]. Other SNPs affecting the destiny of triptans are crucial to their pharmacokinetics and pharmacodynamics. The enzymes responsible for triptan degradation monoamine oxidase (MAO) A and the cytochrome CYP1A2 influence the response to triptans [25] and the SNP rs4680 of the catechol-O-methyltransferase (COMT) increases the risk of poor response to frovatriptan [26]. Therefore, SNPs occurring along the CGRP receptor pathway could affect the clinical evolution of migraine and, thus, might influence to some extent the responsiveness to anti-CGRP(R) mAbs [20]. In particular, for mAbs directed towards CGRP and its receptor the affinity for the functional receptor effectors Fc receptors is fundamental for the maintenance of the antibody-ligand complex and for the mAb elimination half-life [27]. FcγRs are responsible for antibody-dependent cell-mediated cytotoxicity (ADCC) in cancer therapy [28, 29], e.g. rituximab. In fact, they can influence the affinity between the FcγRs and the IgG [30] and the alteration of this mechanism can be of fundamental importance for the effectiveness of anti-CGRP/CGRP(R) mAbs. Polymorphisms and gene copy-number variations (CNVs) of FcRs have been associated to the efficacy of mAbs, as it may occur for trastuzumab [31]. Finally, epigenetic modifications, including DNA methylation and post-translational modifications of the histones tails, have been implicated in modulation of attack frequency [32]. The aim of this systematic review is to assess whether there are SNPs or methylation patterns along the CGRP pathway that can influence susceptibility to migraine, with and without aura, frequency and severity of attacks and responsiveness to treatment.

## Methods

### Objectives and protocol

To our knowledge the present systematic review is the first designed to verify the working hypothesis that the SNPs or methylation patterns occurring along the CGRP pathway can affect the clinical features and the course of disease and the responsiveness of patients affected to anti-migraine therapy. In order to address this PICOS (participants/population, interventions, comparisons, outcomes, and study design) question, the PRISMA recommendations [33, 34] and the guidance from the Human Genome Epidemiology Network for reporting gene-disease associations [35] have been followed. The possibility of SNPs to account for some lack of response to anti-CGRP(R) mAbs, mainly, and to anti-migraine therapy, in general, is a broad question still representing a lack of knowledge; therefore, this work will provide an overview of evidence, including all the existing studies investigating direct genetic association, for assessing the consistency of the body of evidence to prompt future research. For this reason, the protocol has not been registered in the International prospective register of systematic reviews PROSPERO. The systematic review and meta-analysis has been conducted in accordance to a protocol established prior to the literature search. The retrieved results have been evaluated and double-checked independently by two researchers. Any conflicts have been resolved by a third author.

### Inclusion criteria

The analysis included genetic association studies assessing the direct genetic association of SNPs or epigenetic modifications affecting genes involved in the CGRP pathway on the following aspects: susceptibility to migraine, with and without aura; frequency and severity of attacks; responsiveness to treatment. No filters about study duration or follow-up and no restrictions concerned with publication date have been applied. *In vitro* and *in vivo* animal studies, narrative or systematic reviews and meta-analysis, abstracts and congress communications, proceedings, editorials and book chapters as well as studies not available in full text and not published in English have been excluded from the analysis.

### Information sources

The literature search has been performed consulting the most relevant scientific databases, i.e. PubMed/MEDLINE, Scopus, Web of Science, the Human Genome Epidemiology (HuGE) Published Literature database (Public Health Genomics Knowledge Base) and Clinicaltrials.gov. The search could not be conducted also on Embase since it was not freely/institutionally available. No restriction of publication date has been applied. The databases have been searched for records matching the search strings used from their inception to May, 21 2021 that was the date of last search.

### Search strategy

The following Medical Subject Headings (MeSH) terms and modifications have been used as search terms in combination: “migraine”, “CGRP”, ‘‘calcitonin gene-related peptide’’, “CGRP receptor”, “CALC A”, “CALC I”, “RAMP 1”, “CALCRL”, “CLR”, “RCP”, “AMYLIN-1”, “AMYLIN-1 receptor”, “polymorphisms”, “SNP(s)”, “epigenetic”, “methylation”, “anti-CGRP(R) monoclonal antibodies”, “anti-CGRP(R) mAbs”, “triptans”, “gepants”, “ditans”.

### Studies selection

The assessment of the inclusion and exclusion criteria and the determination of eligibility of the studies has been carried out independently by two authors for minimizing the risk to exclude relevant records. Duplicate records have been eliminated and the following first screening has assessed the title and abstract. Then, the full text has been evaluated for inclusion in qualitative and/or in quantitative synthesis. The references list of the articles has been evaluated in order to extend and refine the search. Complete consensus among all the authors has been achieved without relevant conflicts planned to be solved through the Delphi method [36].

### Data analysis

The synthesis of the results has been conducted according to the Cochrane Consumers and Communication Review Group guidelines [37]. The assessment of the risk of bias and of the quality of retrieved studies has been performed in agreement to Human Genome Epidemiology (HuGE) systematic reviews and meta-analyses risk-of-bias score for genetic association studies [38]. Hence, the latter score ranges from low to unclear and high risk, taking into account the following 4 outcomes rated yes, no or unclear: (1) Information bias – Accuracy of diagnosis of migraine and robustness of genotyping methods; (2) Confounding bias – Population stratification and other confounder effects; (3) Selective reporting of outcomes – reporting bias; (4) Hardy-Weinberg equilibrium (HWE) – assessment in the control groups. Sample size has been considered. Due to the heterogeneity of the studies and the difference of polymorphisms and outcomes investigated in the studies meeting the eligibility criteria, data concerned with odds ratios for genotypes could not be pooled and a genotype-based meta-analysis and assessment of the credibility of cumulative evidence through the Venice guidelines [39] have not resulted feasible.

## Results

### Selection of the studies

The search on the databases has retrieved 800 results: 396 records have been obtained form PubMed/MEDLINE, 209 from Scopus, 173 from Web of Science, 19 from the HuGE Published Literature database (Public Health Genomics Knowledge Base) and 3 from Clincaltrials.gov. The 800 records have been searched for duplicates. After duplicates removal there were 285 results to screen (also one of the three records obtained from Clincaltrials.gov has resulted to be a duplicate). The latter have been screened in title and abstract leaving 11 results to assess for eligibility. However, the study by An et al., 2017 [40] was not available in full text and the two records retrieved from Clincaltrials.gov are two studies without results since one is recruiting and the other not yet. In particular, the study INTERROGATE, Biomarker and Genetic Predictors of Erenumab Treatment Response (NCT04265755) is in the recruitment stage and it aims at exploring the relationship between clinical response to erenumab and genetic biomarkers, while the purpose of the BIOmarkers of MIGraine (BIOMIGA) proof of concept study (NCT04503083) is to detect biomarkers predictive of response to anti-CGRP(R) mAbs in severe migraineurs using, among others, pharmacogenetic evaluation and assessment of the methylation levels. Therefore, the full-text articles assessed for eligibility are 8 and 7 of them have met the inclusion criteria for qualitative analysis. In fact, the study by Louter et al., that considered the polymorphisms rs2956 of CALC A gene and rs858745 of CALCRL gene possible candidate genes to be implicated in chronification [41] had to be excluded because of its different study design being a three stage genetic association study.

**Fig. 2:**
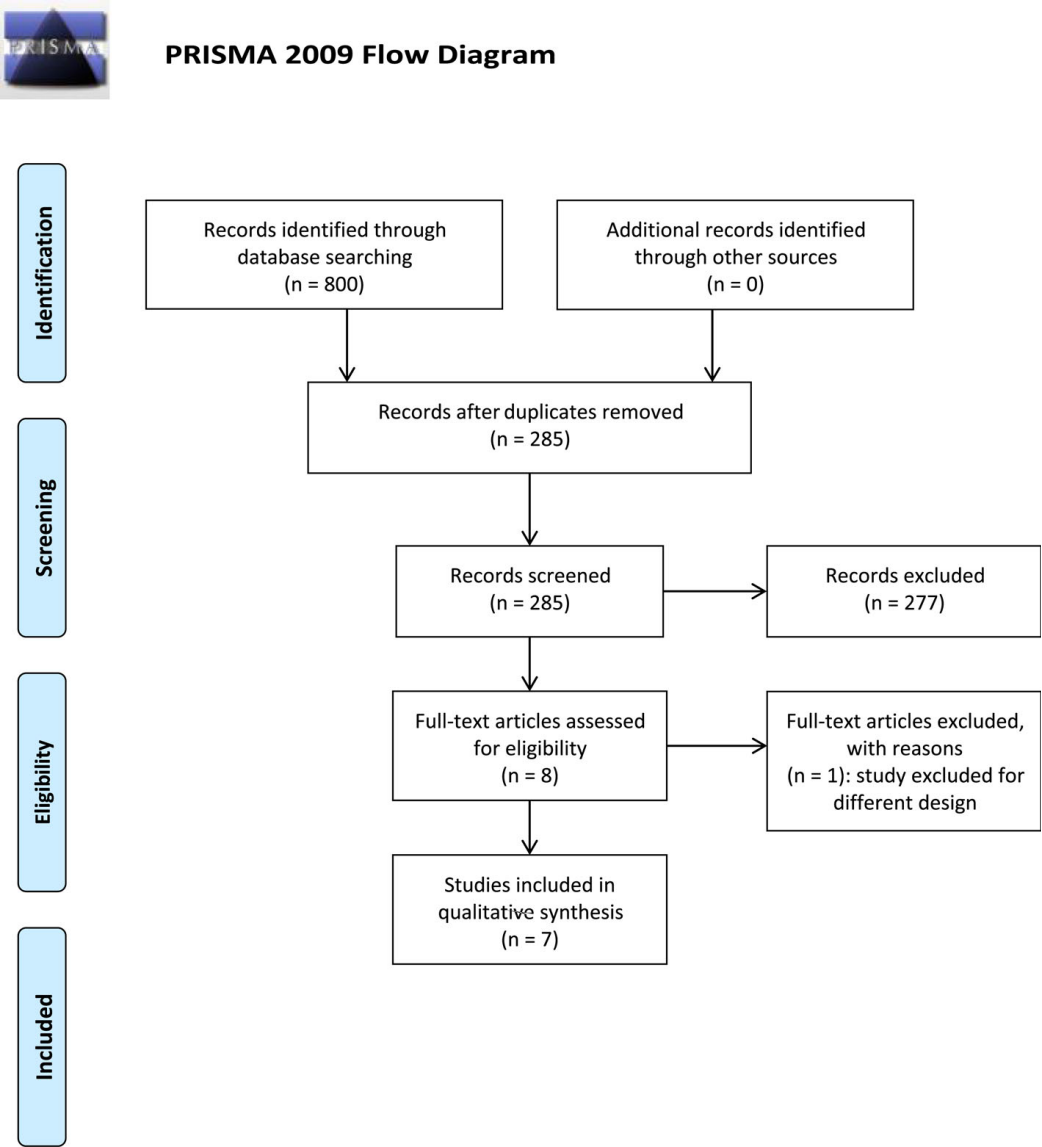
PRISMA flow diagram. PRISMA flow diagram reporting the process of identification and selection of the studies eligible for the systematic review and meta-analysis.

The process of identification and selection of the studies is illustrated in Fig. 2.

### Qualitative analysis

The 7 articles eligible for analysis are grouped according to the gene of which the polymorphisms have been studied, based on the Cochrane Consumers and Communication Review Group guidelines. A summary of the main characteristics of the studies is reported in Table 1.

**Table 1:** Main characteristics of the studies included in the analysis. General characteristics of studies included in the analysis. OR = odds ratio; CI = confidence interval; *P* = P value; HWE = Hardy–Weinberg equilibrium.

Study	Design	Disease (N)	Control (N)	Ethnicity	Sample	Variant	Outcome	Genotype 1	Genotype 2	Genotype 3	HWE	Results
Lemos et al., 2010 [42]	Case-control study. The Authors state that sample size is a limitation of the study not providing enough power to detect a variant with an OR < 1.5.	Diagnosis according to the International Headache Society (IHS), using the International Classification of Headache Disorders (ICHD) I (before 2004) and II. *N* = 188	People with no history of migraine, age-matched to cases *N* = 287	Portuguese	Genomic DNA extraction from peripheral blood leucocytes	rs1553005 (CGRP)	1) Investigate the role of BDNF in migraine susceptibility; 2) CGRP, studied for the first time as candidate gene in migraine; 3) possible interaction of BDNF and CGRP genes in migraine’s susceptibility.	GG: OR (95 %CI) 1.00 0), *p*= -; multivariable logistic regression analysis	GC: OR (95 %CI )1.40 (0.94–2.08), *p* = 0.09; multivariable logistic regression analysis	CC: OR (95 %CI) 0.86 (0.42–1.77), *p* = 0.69; multivariable logistic regression analysis	Case and control groups in HWE for the polymorphism	rs1553005(CGRP)*rs2049046(BDNF) = GC*AT – OR 1.88 (95 %CI 1.20–2.93), *P* = 0.005
Menon et al., 2011 [43]	Case-control study. Power analysis reported	Clinical diagnosis by an experienced clinical neurologist based on international criteria *N* = 278	Individuals with no family history of migraine, age, sex and ethnicity matched to cases *N* = 322	Australian (east coast) Caucasian:of European descent living in Australia, with ancestors emigrated within the last 160 years from the British Isles and other parts of Europe	Genomic DNA extraction from white blood cells	rs35815751 (16 bp deletion in intron 1 of the CALC A gene)	Invetigate the role of 16 bp deletion in the first intron of the CALCA gene in the risk of migraine	II (homozygous, insertion/insertion); no deletion/deletion OR (95 %CI) 1.2 (0.74–1.85) in migraine, for genotypes (*P* = 0.575) and for alleles (*P* = 0.502); logistic regression analysis	ID (heterozygous, insertion/deletion); no deletion/deletion OR (95 %CI) 1.1 (0.68–1.79) in migraine with aura, (genotypes, *P* = 0.666; alleles, *P* = 0.7); logistic regression analysis	DD (homozygous, deletion/deletion); no deletion/deletion OR (95 %CI) 1.6 (0.70–3.44) in migraine without aura, (genotypes, *P* = 0.325; alleles, *P* = 0.276); logistic regression analysis	Genotypes in HWE (migraineurs: P_HWE_=0.73 and controls: P_HWE_=0.247)	No association between rs35815751 and migraine for genotypes (*P* = 0.575) nor alleles (*P* = 0.502), and migraine with aura (genotypes, *P* = 0.666; alleles, *P* = 0.7) or without aura (genotypes, *P* = 0.325; alleles, *P* = 0.276)
Guldiken et al., 2013 [44]	Case-control study on females.	Diagnosis of migraine by neurologist based on the criteria of ICHD-II. *N* = 134	Healty volunteers, health care personal and postpartum females who were hospitalized in the obstetric clinic. *N* = 96	Local (Turkish)	DNA isolation from peripheral blood	rs3781719 (SNP T-692 C of CALC A gene)	Investigated the frequency of CALCA T-692 C in migraineurs and its association to migraine attack frequency and severity	TT: 42.5 %	TC: 42.5 %	CC: 14.9 %	HWE has been evaluated.	No association between the genotype and allele frequency of migraine (*P* = 0.44), without and with aura (*P* = 0.52), and the severity and frequency of migraine attacks. Frequency of migraine attacks has been measured as the number of attacks in a month. The severity has been assessed with the visual analog scale
Sutherland et al., 2013 [45]	Case-control study. Power analysis reported	Diagnosis by a clinical neurologist according to the IHS criteria. *N* = 284	Matched for sex, age (+/−5 years) and ethnicity. *N* = 284	South Eastern Australian: adult Caucasians of European descent living in Australia, with ancestors emigrated within the last 160 years from British Isles and other parts of Europe	DNA extraction from white blood cells	rs3781719 (SNP 624 (T/C) of the CALC A gene promoter)	Investigate the contribution of the 4 selected SNPs in the risk of migraine	TT: -migraine47.8 %; -migraine with aura 49.0 % -migraine without aura 45.7 %	TC: -migraine44.7 %; -migraine with aura 42.2 %; -migraine without aura 49.4 %	CC: -migraine7.5 %; -migraine with aura 8.8 %; -migraine without aura 4.9 %	HWE has been verified. Genotypes for SNPs were in HWE in case and control groups	No significant association with migraine (*P* = 0.260), migraine with aura (0.563) and migraine without aura (0.133)
						rs145837941 (4218T > C base-exchange in the coding sequence of CALC A)		TT: 94.9 %	TC: 4.7 %	CC: 0.4 %		No significant association with migraine (*P* = 0.913)
						rs3754701 (SNP in the RAMP1 gene promoter at position − 1166 (T/A))		TT: - migraine 37.4 %; - migraine with aura 42.1 %; - migraine without aura 30.6 %	TA: - migraine 49.4 %; - migraine with aura 44.8 %; - migraine without aura 56.1 %	AA: - migraine 13.2 %; - migraine with aura 13.1 %; - migraine without aura 13.3 %		No significant association with migraine (*P* = 0.360), migraine with aura (*P* = 0.276) and migraine without aura (*P* = 0.260)
						rs7590387 ((G/C) 1.4 kb downstream of the RAMP1 gene)		GG: - migraine 25.7 %; - migraine with aura 23.9 %; - migraine without aura 28.4 %	GC: - migraine 54.4 %; - migraine with aura 54.3 %; - migraine without aura 54.5 %	CC: - migraine 19.9 %; - migraine with aura 21.7 %; - migraine without aura 17.0 %		No significant association with migraine (*P* = 0.341), migraine with aura (*P* = 0.566) and migraine without aura (*P* = 0.299)
Cargnin et al., 2015 [46]	Case-control study. Power calculation reported	Diagnosis fulfilling ICHD-II criteria for migraine without aura A for at least 1 year and for medication overuse headache (MOH) or previous or current diagnosis of MOH.Patients with migraine without aura and patients with MOH. N (migraine without aura) = 219; N (MOH) = 130	Matched by age and sex and of same ethnicity. *N* = 209	Italian (north-west)	DNA extraction from peripheral blood	rs3781719 [CALC A (T > C)]	Investigate the role of the two selected SNPs in: 1) risk of inconsistent response to triptans; 2) risk of trasformation of episodic migraine to MOH	TT: (A) OR (95 %CI) 0.96 (0.62–1.48), *P* = 0.84; logistic regression analysis adjusted for triptan (frovatriptan vs. other triptans), and for polymorphisms, associated with triptan response. A = log-additive model of inheritance; D = dominant model of inheritance; R = recessive model of inheritance	TC: (D) OR (95 %CI) 1.04 (0.59–1.85), *P* = 0.88; logistic regression analysis adjusted for triptan (frovatriptan vs. other triptans), and for polymorphisms, associated with triptan response. A = log-additive model of inheritance; D = dominant model of inheritance; R = recessive model of inheritance	CC: (R) OR (95 %CI) 0.80 (0.30–2.11), *P* = 0.65; logistic regression analysis adjusted for triptan (frovatriptan vs. other triptans), and for polymorphisms, associated with triptan response. A = log-additive model of inheritance; D = dominant model of inheritance; R = recessive model of inheritance	HWE tested. P_HWE_ rs3781719 = 0.52; P_HWE_ rs3754701 = 0.77; P_HWE_ rs7590387 = 0.41. For only MOH patients: (P_HWE_ rs3781719 = 0.18; P_HWE_ rs3754701 = 1; P_HWE_ rs7590387 = 0.69)	No significant association for endpoint 1 [risk of inconsistent response to triptans (being consistent responders defined as patients experiencing a ≥ 2 point reduction after triptan administration in at least 2 out of 3 consecutive attacks)]; significant association of RAMP1rs7590387GG [OR (95 %CI) (R) 0.27 (0.13–0.57) *P* = 0.0002)] for endpoint 2 (risk of trasformation of episodic migraine to MOH)
						rs3754701 [RAMP 1 (T > A)]		TT; (A) OR (95 %CI) 0.90 (0.60–1.37), *P* = 0.63; logistic regression analysis adjusted for triptan (frovatriptan vs. other triptans), and for polymorphisms, associated with triptan response. A = log-additive model of inheritance; D = dominant model of inheritance; R = recessive model of inheritance	TA; (D) OR (95 %CI) 0.73 (0.41–1.32), *P* = 0.30; logistic regression analysis adjusted for triptan (frovatriptan vs. other triptans), and for polymorphisms, associated with triptan response. A = log-additive model of inheritance; D = dominant model of inheritance; R = recessive model of inheritance	AA; (R) OR (95 %CI) 1.19 (0.53–2.67), *P* = 0.66; logistic regression analysis adjusted for triptan (frovatriptan vs. other triptans), and for polymorphisms, associated with triptan response. A = log-additive model of inheritance; D = dominant model of inheritance; R = recessive model of inheritance		
						rs7590387 [RAMP 1 (C > G)]		CC: (A) OR (95 %CI) 1.11 (0.75–1.65), *P* = 0.60; logistic regression analysis adjusted for triptan (frovatriptan vs. other triptans), and for polymorphisms, associated with triptan response. A = log-additive model of inheritance; D = dominant model of inheritance; R = recessive model of inheritance	CG: (D) OR (95 %CI) 0.84 (0.46–1.54), *P* = 0.57; logistic regression analysis adjusted for triptan (frovatriptan vs. other triptans), and for polymorphisms, associated with triptan response. A = log-additive model of inheritance; D = dominant model of inheritance; R = recessive model of inheritance	GG: (R) OR (95 %CI) 1.69 (0.86–3.30), *P* = 0.12; logistic regression analysis adjusted for triptan (frovatriptan vs. other triptans), and for polymorphisms, associated with triptan response. A = log-additive model of inheritance; D = dominant model of inheritance; R = recessive model of inheritance		
Wan et al., 2015 [48]	Exploratory analysis. Limitations reported by the Authors: small sample, the analysis could not be conducted on the specific tissue derived from trigeminovascular or cerebral biopsies,the blood DNA was from multiple cell lineages with likely different DNA methylation	Migraineurs recruited at International Headache Center of Chinese PLA General Hospital. *N* = 26	Matched healthy controls with no significant differences in gender and age compared to migraineurs group. *N* = 25	Patients recruited at International Headache Center of Chinese PLA General Hospital	DNA extraction from EDTA blood samples obtained through the cubital vein	Analysis of CpG islands of the RAMP1 gene in a 3000 bp region including putative promoter sequences and the first exon	Investigate if the methylation pattern of the promoter of RAMP1 gene in peripheral leukocyte is associated with migraine	Low methylation trend without statistical significance in migraine vs. control (total average methylation level: 8.41 % ±1.92 % vs. 9.90 % ± 3.88 %, *P* = 0.197)			The ROC the curve and Youden criterion have been used for the determination of the optimum cut-off of methylation level	No significant difference in the DNA methylation pattern of RAMP 1 between migraine and control groups
Moreno-Mayordomo et al., 2019 [47]	Prospective, observational, multicentre, study enrolling female participants. Sample power calculation reported	Diagnosis of chronic migraine according to ICHD- III edition, beta version. classified as responders to onabotulinum toxinA (defined as defined as a reduction of at least 50 % in the number of monthly migraine days 3 months after the second administration. *N* = 117	Diagnosis of chronic migraine according to ICHD- III edition, beta version. classified as non responders to onabotulinum toxinA. *N* = 33	Caucasian ethnicity and Spanish origin	Genomic DNA extraction from peripheral blood anticoagulated in EDTA-K_3_	rs3781719 (CALC A)	Investigate the effect of rs3781719 on the response to OnabotulinumtoxinA	TT: 53.85 % responders and 27.27 % non responders	TC: 38.46 % responders and 63.63 % non responders	CC: 7.69 % responders and 9.09 % non responders	All of the variants wer in HWE Apart from rs222749 of TRPV 1 gene.	Significant differences in risk to lack of response to onabotulim toxin A for the SNP rs3781719 of gene CALC A: OR (95 %CI) (dominant model) 3,11 (1,33 − 7,26), (codominant model) 1,6 (0,85 − 3,0), (recessive model) 1,2 (0,31 − 4,71)

### Polymorphisms of the gene encoding CGRP

The gene encoding CGRP has been studied for the first time in the study by Lemos et al., in a European population [42]. The SNP rs1553005 of the latter gene has been found to interact with the variant rs2049046 of the gene encoding brain-derived neurotrophic factor (BDNF) increasing the risk of migraine [rs1553005(CGRP)*rs2049046(BDNF) = GC*AT – OR 1.88 (95 %CI 1.20–2.93), *P* = 0.005] [42]. This is supported by the co-expression of the latter neurotransmitter that has been demonstrated in trigeminal ganglion neurons of rat. The study by Menon and collaborators [43] has investigated in an Australian population the role of the polymorphism rs35815751, consisting in a 16 bp deletion in the first intron of the CALC A gene that is a region with triplet G-run motifs, in the development of migraine with aura: no significant association between rs35815751 and migraine [for genotypes (*P* = 0.575) nor alleles (*P* = 0.502)], and migraine with aura (genotypes, *P* = 0.666; alleles, *P* = 0.7) or without aura (genotypes, *P* = 0.325; alleles, *P* = 0.276) has been found. The study of Guldiken and collaborators [44] has tested on female population the possible influence of the polymorphism rs3781719, consisting in T-692 C of CALC A gene, on attack frequency and severity and on the occurrence of aura, finding no significant association with migraine (*P* = 0.44) and aura (*P* = 0.52). Also in an Australian population, the study by Sutherland et al., [45] has investigated the possible correlation between the SNPs rs3781719 in the promoter region and rs145837941 in the coding sequence of CALC A and an increased susceptibility to migraine. None of the two polymorphisms have resulted associated to migraine susceptibility or with gender and the rs3781719 has not been associated to increased frequency of attacks or to the development of aura. This polymorphism has been studied also by Cargnin et al., in an Italian population for its influence on response to triptans in patients affected by migraine without aura and it has been tested for association with transformation into MOH, providing no significant correlation [46]. However, CALC A rs3781719C allele has resulted to increase risk of lack of response to OnabotulinumtoxinA in a female population of Caucasian ethnicity and Spanish origin [OR (95 %CI) (dominant model) 3,11 (1,33 − 7,26), (codominant model) 1,6 (0,85 − 3,0), (recessive model) 1,2 (0,31 − 4,71)] in the study by Moreno-Mayordomo and coworkers [47].

### Polymorphisms of the gene encoding RAMP 1

The SNP rs3754701, in the promoter region, and the rs7590387 of the gene encoding RAMP1 have been investigated for the first time by Sutherland and collaborators [45], but no significant associations with migraine susceptibility have been identified. The SNP rs3754701 has been tested also with the SNP rs7590387 of RAMP 1 in migraineurs not presenting aura for association with response to triptans and as risk factors for MOH by Cargnin et al., [46]. Using the log-additive, the dominant and the recessive model of inheritance response to triptans has not been correlated, but the rs7590387G allele and the rs7590387GG genotype reduce significantly the risk of transformation of episodic migraine into MOH [OR (95 %CI) (R) 0.27 (0.13–0.57) *P* = 0.0002)] [46]. Incidentally, in patients affected by migraine a methylation trend (lower in females) at the promoter region of the gene encoding RAMP 1 without significant differences in the DNA methylation level has been detected by Wan et al., [48].

### Assessment of quality of the studies

The quality of the studies included in the present systematic analysis has been assessed following the HuGE systematic reviews and meta-analyses risk-of-bias score for genetic association studies [38] considering the following 4 outcomes: (1) Information bias, evaluating the accuracy of diagnosis of migraine, the ascertainment of controls matched to cases and the quality of genotyping; (2) Confounding bias, in which all the confounders e.g. population stratification, different ethnicity/gender, sample power calculation and statistical adjustment for confounders have been considered; (3) Selective reporting of outcomes, i.e. mentioning only significant associations with SNPs; (4) HWE assessment. The studies by Lemos et al., 2010, Guldiken et al., 2013, Sutherland et al., 2013, Cargnin et al., 2015 and Moreno-Mayordomo et al., 2019 do not present risk of bias since the criteria for diagnosis of cases vs. controls have been clearly stated and have followed the criteria of the IHS and of the ICHD in effect at the time of the study. In the other studies, clinical neurological assessment, also according to international criteria [43], has been reported, originating unclear risk of bias. The ascertainment of sex, age and ethnicity matched controls has been mentioned in the study by Lemos et al., 2010, Menon et al., 2011, Sutherland et al., 2013, Cargnin et al., 2015, Wan et al., 2015. In the study by Moreno-Mayordomo et al., 2019 both responders and non responders have been determined as chronic migraineurs using the ICHD-III edition, beta version. The demographic and clinical characteristics between case and control groups have resulted comparable, or subjected to further subgroup analyses, in the studies by Cargnin et al., Lemos et al., Menon et al., and Moreno-Mayordomo et al. Apart from multiple comparisons, adjusted analyses for confounding effects, e.g. for triptan type or for SLC6A4 STin2 VNTR and COMT val158me polymorphisms associated to triptan response [46], have been conducted in the studies by Cargnin et al., 2015, Moreno-Mayordomo et al., 2019, Menon et al., 2011 and Wan et al., 2015. Weak linkage disequilibrium, indirect genetic association with the true causal variant [49], has been reported in the study by Lemos et al., 2010. Linkage disequilibrium has been performed also in the study by Cargnin et al. and in the study by Sutherland et al. The methods of genotyping have been reported by all the studies. Compromised quality of DNA and successful genotyping have been reported and samples removed from analysis where occurring, e.g. Lemos et al., Menon et al., and Sutherland et al. On the contrary, no discrepancies in genotyping, that had even been re-conducted for validation in about 10 % of the samples, have been found in the study by Cargnin and coworkers. In the study by Guldiken and collaborators smoking and family history of vascular disease were significantly more frequent in the migraine group, but no analysis adjusted for confounders has been reported. In the study by Moreno-Mayordomo and coworkers two of the considered variables have resulted to present significant differences after correction for multiple comparisons between the groups of responders and non-responders. In the study by Cargnin and collaborators, the Authors report that the possibility that some individuals present in the control sample might be affected by MOH cannot be excluded, but that this fraction would be unlikely to be higher than that observed in the general population. The sample power calculation has been reported in the studies by Lemos et al., Menon et al., Sutherland et al., Cargnin et al., Wan et al., and Moreno-Mayordomo et al. However, in the study by Lemos et al., the Authors highlight that sample size is a limitation of the study not providing enough power to detect a variant with an OR < 1.5 and that the study had a power of 64 % to detect an association with the included sample (for a nominal significance level of 0.05). Cargnin et al. report that the study is underpowered to detect small genetic main effects but, its power is sufficient for medium-large effect sizes of clinical relevance. Wan et al. state that sample size calculation could not be accurate since it was the first study investigating RAMP1 methylation pattern and that, therefore, a wider cohort is needed. Other limitations mentioned by Wan et al. are relative to blood DNA sample coming from multiple cell lineages with likely different DNA methylation and the issue concerned with the impossibility to conduct the analysis on the specific tissue derived from trigeminovascular or cerebral biopsies. All the studies are devoid of reporting bias. The HWE has been assessed in all the studies and ROC curve and Youden criterion have been used for the determination of the optimum cut-off of methylation level in the study by Wan et al., but the P_HWE_ values have been reported only in the studies by Cargnin et al. and Menon et al.

### Discussion and conclusions

Interest in migraine and in SNPs likely linked to susceptibility to its development and clinical features has been growing over the last years. However, little is known about the clinical relevance of these polymorphisms and their effect on the response to anti-migraine treatment. Moreover, the new era of migraine preventative treatments has been opened by the anti-CGRP(R) mAbs, but some 40 % non-responders still represent a pharmacological unmet need. This is the first systematic review that intends to identify SNPs affecting different segments of the CGRP pathway and assessing how they can influence migraine from its development, to the presence of aura and to the efficacy and safety of treatment. From an initial screening of the 800 records identified through database searching, only 7 studies met the inclusion criteria. This is the first obvious red flag that SNPs concerned with the CGRP signaling of clinical relevance are poorly investigated. Within these 7 studies for a total of 2413 patients SNPs and methylation affecting CALC A and RAMP 1 genes have been detected and the most recurring is the rs3781719 exchange (T > C), a single nucleotide variation (SNV) originating a 2KB upstream variant in the CALC A gene promoter, of which clinical significance is not determined [50]. From our analysis no significant association has been found between the SNV rs3781719 and the clinical characteristics of migraine manifestation, i.e. increased frequency of migraine, presence of aura and gender differences [45]. Not even any correlations with responsiveness to triptans under the log-additive, the dominant and the recessive model of inheritance have been highlighted [46], but it has been associated with an increased risk of lack of response to onabotulinumtoxinA [51]. These results have been obtained in population of different gender and ethnicity, being Australian [45], Italian [46] and females of Spanish origin [51]. However, from the study by Guldiken and coworkers we have learnt that the rs3781719 T-692 C is not associated to migraine attacks frequency and severity and to the occurrence of aura in Turkish females [52]. Incidentally, the effects of CALC A rs3781719 (-692T > C) have been studied in Chinese Han women with chronic postsurgical pain 6 months after cesarean section. [53]. The rs3781719C allele has been demonstrated to represent a risk factor for this postoperative chronic pain condition [53]. According to our analysis, the second most investigated SNP affecting the CGRP pathway is the rs7590387 of RAMP 1 locus [45, 46], that is another SNV (C > G), also in this case without a demonstrated clinical significance [54]. The latter had been previously investigated in a genome-wide association study finding correlation with migraine [55], but these data had not been published. Although no significant association with migraine susceptibility in general and both with and without aura [45] and with the response to triptans in all the three genotypes under study [46], the rs7590387G allele and the rs7590387GG genotype have been found to reduce significantly the risk of transformation MOH [46]. Interestingly, the relationship of this SNP with cerebral infarction has been examined in a Japanese population, suggesting the T-A-C haplotype to represent a genetic marker for cerebral infarction [56]. Only one study investigating the methylation pattern with regard to CGRP pathway has resulted from the search. The results of this systematic analysis must be interpreted based on the characteristics of the included studies. In fact, it is important to notice that the genetic variations examined are different within the studies and, also when the same SNP has been investigated, the outcomes considered differed markedly among these studies. Also, the methods of analysis of the results do not make comparison feasible. Moreover, they have been performed on populations not comparable for gender and ethnicity, thus not allowing the generalization of the results. Although the study design is the case-control typical to test for direct genetic associations and the overall quality of the studies meet lot of the HuGe criteria, some concern has been raised: the ascertainment of diagnosis in cases and the matching with controls is not always efficient and clearly reported; the features of migraine considered are different; the adjustement for confounders is not always present and not in each study all the parameters assessing the HWE and efficiency of genotyping are reported completely. Together with the described heterogeneity between the studies, the small sample size and the lack of an adequate number of references for power calculation is a fundamental issue strengthening the need for more investigation in this research field. This is more evident for the exploratory analysis study by Wan et al. Often genetic association studies show initial significant association, but the latter needs to be confirmed by replication studies. These studies must be adequately powered to detect true associations, also in case of small effect conferred by genotype or allele, in order to exclude the risk of false-positive findings arisen by chance or systematic bias; unfortunately, frequently this does not occur [49]. To provide a hypothesis of functional significance of the polymorphisms found in the context of CGRP signalling and migraine, it is mandatory to consider that the data that have resulted close to significance but without reaching it may have been affected by: (1) the small sample size of the cohorts, underpowered to detect small genetic main effects and for further stratification analysis; (2) the confounding influence of environmental factors. However, the results of this systematic review suggest a role of rs7590387GG of RAMP1 in the transformation of episodic migraine into MOH. Interestingly, the collected data have highlighted that rs3781719 of gene CALC A influences the response to onabotulinumtoxinA. Moreover, the first comprehensive genetic association study of patients with chronic and high-frequency migraine has underscored rs2956 of CALC A gene and rs302680 of RAMP1 to be nominally associated with chronic migraine, although these associations have not resulted significant in the replication stage [57]. Therefore, a role of genetic variants affecting the CGRP pathway and, thus the responsiveness to therapeutics, emerges from this systematic review and it warrants further investigation. Remarkably, no genetic association studies investigating the possible effect of CGRP SNPs on the responsiveness to the most recent pharmacological approaches, i.e. anti-CGRP(R) mAbs, gepants and ditans, have been found, apart from the ongoing INTERROGATE trial. The latter mAbs are a fundamental weapon in the arsenal of migraine therapy even more for difficult-to-treat patients who do not find relief from treatment. In fact, the efficacy of mAbs targeting CGRP in refractory patients has provided very encouraging results [58]. Furthermore, these mAbs are well tolerated and titres of neutralizing and not anti-mAbs antibodies that have been reported were low and not affecting significantly efficacy and safety [58, 59]. Therefore, the existence of clinically relevant genetic variants along the CGRP signaling deserves further investigation since these could account for some percentage of non responders to treatment with mAbs. In addition, several conditions may lead to lack of responsiveness with consequent persistent migraine [60]: it is possible that CGRP is not completely inhibited in its action or that, although fully blocked, adrenomedullin can still induce vasodilation through heteromerization of its receptor with CGRP(R). Furthermore, the role of other peptides, e.g. pituitary adenylate cyclase-activating polypeptide (PACAP) or the vasoactive intestinal peptide (VIP) deserves investigation [60]. An attempt to predict the responders to erenumab has highlighted that a lower baseline mean blood flow velocity in cerebral arteries is associated to increased effectiveness, with cerebral blood flow increase after treatment in good responders [61]. Incidentally, early non responders to galcanezumab can turn into responders in the second/third month [62]. Also, recently an association between iron deposits in the periaqueductal gray of patients suffering from chronic migraine and poor response to onabotulinumtoxinA has been observed [63], pointing at the possible involvement of metal deposits in the responsiveness to anti-migraine treatments. Thus, it is fundamental to understand how SNPs and epigenetic modifications may affect the response to anti-migraine treatment, since most drugs directly or indirectly target the latter pathway, in order to predict treatment efficacy and safety. A window is opening on the association between disabling primary headache and preclinical familial Alzheimer’s disease [64]. Moreover, both dementia and migraine are main symptoms of CADASIL (cerebral autosomal dominant arteriopathy with subcortical infarcts and leukoencephalopathy, OMIM#125,310) [65]. Therefore, the genetic association of these widespread disabling diseases needs to be studied. Furthermore, aged populations need to be included in migraine genetic association studies, as well as in clinical trials for migraine  treatment [66–71]. Hence, adequately powered studies following the criteria for rigorous genetic association are needed to provide high quality evidence on the impact of genetic variants affecting CGRP signaling on migraine susceptibility and clinical manifestation and to predict the response to therapy in terms of efficacy and safety.

## Data Availability

All data generated or analysed during this study are included in this published article/Data sharing is not applicable to this article as no datasets were generated or analysed during the current study.

## Abbreviations

ACE: angiotensin converting enzyme
AV: arterial vessels
BIOMIGA: BIOmarkers of MIGraine
CALCRL: human G protein-coupled receptor calcitonin receptor-like receptor
CDEM: chronic disorders with episodic manifestations
CI: confidence interval
CGRP: calcitonin-gene related peptide
COMT: catechol-O-methyltransferase
CSD: cortical spreading depression
Glu: glutamate
HLA: human leukocyte antigens
HuGE: Human Genome Epidemiology
HWE: Hardy–Weinberg equilibrium
mAbs: monoclonal antibodies
MAO: monoamine oxidase
MTHFR: methylenetetrahydrofolate reductase
MTR: methionine synthase
MTRR: methionine synthase reductase
NO: nitric oxide
OR: odds ratio
PICOS: participants/population, interventions, comparisons, outcomes, and study design
PRISMA: Preferred Reporting Items for Systematic reviews and Meta-Analyses
PROSPERO: International prospective register of systematic reviews
RAMP-1: receptor activity-modifying protein
SNP: single-nucleotide polymorphism
SNPs: single-nucleotide polymorphisms
YLD: Years Lived with Disability
